# β7 integrins contribute to intestinal tumor growth in mice

**DOI:** 10.1371/journal.pone.0204181

**Published:** 2018-09-20

**Authors:** Srustidhar Das, Cristian Doñas, Paulina Akeus, Marianne Quiding-Järbrink, J. Rodrigo Mora, Eduardo J. Villablanca

**Affiliations:** 1 Department of Medicine, Solna, Science for Life Laboratory, Karolinska Institute and University Hospital, Stockholm, Sweden; 2 Department of Microbiology and Immunology, Institute of Biomedicine, the Sahlgrenska Academy at the University of Gothenburg, Gothenburg, Sweden; 3 Massachusetts General Hospital, Harvard Medical School, Boston, MA, United States of America; 4 Takeda Pharmaceuticals, Cambridge, MA, United States of America; University of New Mexico, UNITED STATES

## Abstract

The gut homing receptor integrin α4β7 is essential for the migration of pro-inflammatory T cells into the gut mucosa. Since intestinal neoplasia has been associated with chronic inflammation, we investigated whether interfering with gut-homing affects intestinal tumorigenesis. Using chemically induced and spontaneous intestinal tumor models we showed that lack of β7 integrin significantly impairs tumor growth without affecting tumor frequencies, with a mild translatable effect on overall survival. This correlates with human data showing lower MAdCAM-1 expression and disease-free survival in colorectal cancer patients. Thus, paradoxically in contrast to extra-intestinal tumors, blocking migration of immune cells into the gut might have a positive therapeutic effect on intestinal neoplasia.

## Introduction

Colorectal cancer (CRC) is the second most common cancer in women and the third most common in men around the world [1] [2]. Among potential causes, intestinal inflammation is associated with a significantly increased risk of developing colorectal cancer [3], and non-steroidal anti-inflammatory drugs decrease the incidence of intestinal cancer in mice and humans [4–6]. In agreement, inflammatory bowel disease (IBD) patients have an increased risk to develop CRC, likely due to elevated inflammatory cytokines [7]. In addition, CD25^+^ Foxp3^+^ regulatory T cells (T_REG_) can prevent the development of inflammation and intestinal cancer in two murine models [8, 9], suggesting that intestinal inflammation plays a key role in the pathogenesis of intestinal neoplasia. In agreement, tumor infiltrating FOXP3^+^ Treg density in human colorectal cancer was shown to be associated with improved survival compared to CD8^+^ and CD45RO^+^ lymphocytes [10].

T effector cells (T_EFF_) continuously monitor the body to detect and destroy neoplastic cells, a process known as tumor immunosurveillance. However, while T_EFF_ are typically protective against tumors in peripheral tissues, they paradoxically promote inflammation-driven neoplasia in the gut [11, 12]. On the other hand, T_REG_ which are conventionally immunosuppressive T cells, by virtue of their anti-inflammatory properties, might prevent and even reverse tumorigenesis in the gut, presumably by dampening inflammation. Thus, the gut mucosa represents a paradox regarding tumor immunosurveillance, as observed in IBD patients who exhibit higher risk of developing colorectal cancer [3, 13].

Given that pro-inflammatory T_EFF_ cells (Th_1_ and Th_17_) require the gut-homing receptor α4β7 to migrate to the gut mucosa [14], we sought to investigate whether α4β7 is required for tumorigenesis. Using two intestinal tumor models we show that β7 integrins are not required for tumorigenesis, but lack of these integrins have a clear impact on tumor size. The involvement of β7 integrins in intestinal tumor growth suggests that blocking gut-homing receptors could be an adjuvant strategy to prevent and/or treat intestinal neoplasia in the context of chronic gut inflammation, as in IBD patients.

## Materials and methods

### Mice

Itgb7^-/-^, C57BL/6, and Apc^Min/+^ (C57BL/6J-APC^Min^/J) mice were purchased from Jackson Laboratories. Apc^Min/+^;Itgb7^-/-^ were generated by breeding Apc^Min/+^ and Itgb7^-/-^. Mice were maintained in SPF/VAF animal facilities at Massachusetts General Hospital (MGH) and University of Gothenburg. This study was carried out in strict accordance with the recommendations in the Guide for the Care and Use of Laboratory Animals of the Subcommittee on Research Animal Care at MGH and Harvard Medical School and the government animal ethics committee at the University of Gothenburg, respectively. The protocol was approved by the Committee on the Ethics of Animal Experiments of the MGH (Ethical permit number 2007N000115) and University of Gothenburg (Ethical permit number 84–2014). All efforts were made to minimize animal suffering.

### DSS-induced colon tumor progression

**Fig 1:**
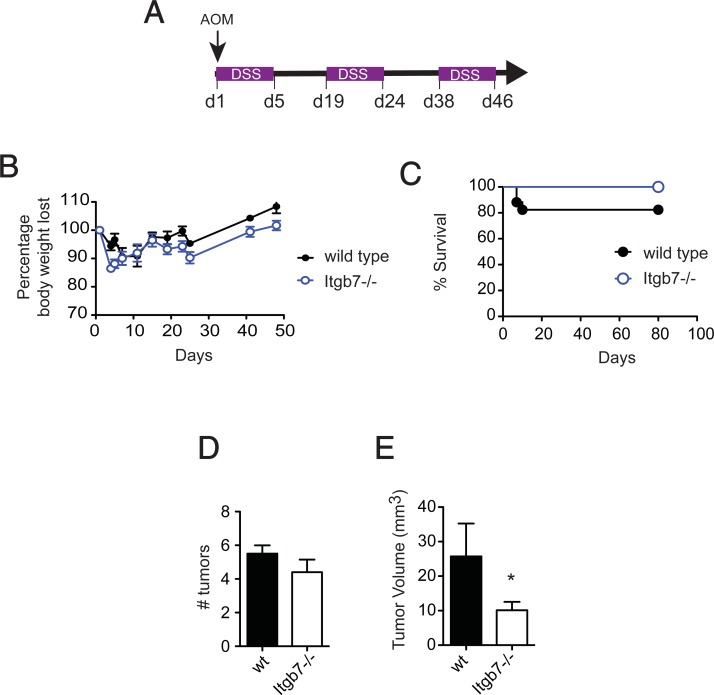
Mice lacking β7 integrins (Itgb7^-/-^) exhibit impaired tumor development in a chemical/inflammation-driven colon tumorigenesis model. (A) Colitis-induced tumorigenesis: AOM (10 mg/kg body weight) was injected i.p. at day 1 followed by 3 cycles of 2.5% DSS. (B) Weight loss of wild type (wt) and Itgb7^-/-^ mice (*n =* 8–14). (C) Kaplan-Meier plot of wt and Itgb7^-/-^ mice survival (*n =* 8–14). Macroscopic tumors were counted (D) and tumor volume measured (E) after 10 weeks (*n* = 8). Mean ± SEM, *p<0.05.

Wild type or Itgb7^-/-^, mice received one intraperitoneal injection of AOM (Sigma Aldrich) at 10 mg/kg body weight followed by 3 cycles of DSS treatments (Fig 1). Mice were sacrificed and tumor analysis were done 10 weeks after AOM injection, corresponding to day 10 after 3 cycles of DSS treatment or after initial signs of distress, such as more than 15% of body weight loss, dehydration or hunched posture.

### Competitive homing experiments

Total T cells isolated from spleen of wild type (CD45.1^het^) were mixed with the congenic Itgb7^-/-^ counterpart (CD45.1^homo^) in a 1:1 ratio and then adoptively transferred intravenously into 10 weeks old Apc^Min/+^ recipients (CD45.2). Mice were euthanized after 12 hours and single cell suspensions from the spleen, small intestine lamina propria (SILP) and intestinal tumors were obtained for analysis. Cell samples were stained to detect T cells (CD3), live cells and congenic markers (CD45.1 and CD45.2) and analyzed on a fluorescence-activated cell sorter (FACS) LSRII (BD Biosciences). Analysis and homing index calculation was performed as described elsewhere [15].

### Transcript analysis in CRC patients

To investigate the relation between MAdCAM-1 mRNA expression and CRC patient survival we used the “R2: Genomics Analysis and Visualization Platform” (http://r2.amc.nl), which contains genome-wide gene expression data from human diseases. The Kaplan curve with gene expression was automatically displayed using the “Tumor Colon CIT (Combat)–Marisa 566 rma–u133p2” dataset and the MADCAM1 (208037_s_at) probe with an expression cutoff of 36.2 (min.grp = 8).

### Statistical analysis

Data are presented as mean ± SEM and were analyzed using GraphPad Prism Software 5.0b. Statistics were calculated using either unpaired *t* test when comparing two groups or ANOVA with Dunnett’s or Bonferroni’s post-hoc test when comparing more than 2 groups. Significance was set at p < 0.05.

## Results and discussion

To investigate whether intestinal tumor development requires gut-tropic immune cells we performed the inflammation-driven AOM/DSS model of colon tumorigenesis (Fig 1A) [16] in wild type (wt) or β7 integrin chain (Itgb7)-deficient mice, which lacks both β7 integrins (α4β7 and αEβ7) [17, 18]. AOM/DSS-induced tumors exhibit high levels of CD3^+^ cell infiltration [19], suggesting that lymphocyte migration to the gut might play a role in tumor progression. Both wt and Itgb7^-/-^ mice showed a similar decrease in body weight upon AOM/DSS treatment and repeated DSS exposure (Fig 1B). Of note, body weight recovery was similar between wt and Itgb7^-/-^ mice during the water cycles, and although Itgb7^-/-^ showed a tendency to lose more weight upon DSS exposure, all Itgb7^-/-^ mice survived for the length of the study. By contrast, 20% of wt mice were euthanized due to signs of marked distress (Fig 1C).

Treatment with AOM/DSS triggered tumorigenesis in all wt and Itgb7^-/-^ mice analyzed (*n* = 8–14), and by week 10 tumor numbers were comparable in wt (5.5 ± 0.7) and Itgb7^-/-^ (4.4 ± 1.6) mice (Fig 1D). However, tumor mass was significantly less in Itgb7^-/-^ compared to wt mice (Fig 1E), and the average tumor volume per mouse was 25 ± 10.7 mm^3^ in wt versus 9.9 ± 6.1 in Itgb7^-/-^ mice. Taken together, our data suggest that gut-tropic immune cells are not required for tumor initiation, but they might promote tumor growth in the context of inflammation-driven intestinal tumorigenesis.

**Fig 2:**
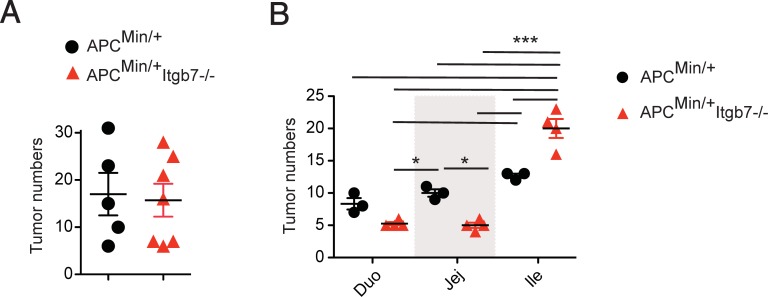
Altered tumor distribution in Apc^Min/+^;Itgb7^-/-^ mice. (A) Apc^Min/+^ and Apc^Min/+^;Itgb7^-/-^ mice were euthanized between 20–25 weeks, the small bowel was dissected, open longitudinally, and mounted for macroscopic tumor analysis. (A) Number of tumors throughout the small bowel length (*n* = 3–4 mice). (B) The small bowel was divided into duodenum (Duo), jejunum (Jej) and ileum (Ile) and macroscopic tumors were counted (*n* = 3–4). Mean ± SEM, ANOVA with Bonferroni as post-test *p<0.05, ***p<0.001.

*Apc* is the most commonly mutated gene in both familiar and sporadic human colorectal cancer [20], making Apc^Min/+^ mice a relevant translational model to study intestinal tumorigenesis. We then asked whether lack of β7 integrins affect tumor development in the Apc^Min/+^ spontaneous model of intestinal tumorigenesis. Consistent with our previous results using a chemically-induced inflammation-driven tumorigenesis model, we observed that Apc^Min/+^;Itgb7^-/-^ mice exhibit similar numbers of intestinal tumors in the whole small bowel when compared to Apc^Min/+^ mice (Fig 2A). However, we observed some regional differences, with lower tumor numbers in the duodenum and jejunum of Apc^Min/+^;Itgb7^-/-^ mice, but increased tumor numbers in the ileum when compared to Apc^Min/+^ mice (Fig 2B).

**Fig 3:**
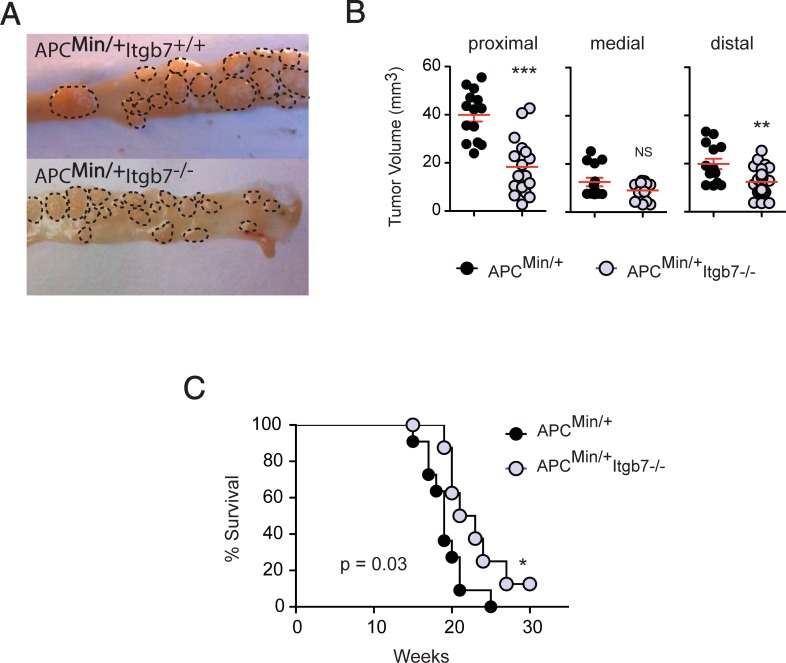
Decreased tumor size in Apc^Min/+^;Itgb7^-/-^ mice. Apc^Min/+^ and Apc^Min/+^;Itgb7^-/-^ mice were euthanized between 20–25 weeks and the small bowel was dissected and open longitudinally for macroscopic tumor analysis. (A) Dashed line delineates single tumors within the small bowel (smaller tumor areas in Apc^Min/+^;Itgb7^-/-^ mice). Pictures are representative of two independent experiments. (B) Tumor volume measured in the duodenum (proximal), jejunum (medial) and ileum (distal). (C) Kaplan-Meier plot of Apc^Min/+^ and Apc^Min/+^;Itgb7^-/-^ mice survival (*n =* 10 mice/group, 3 experiments). Data are from three independent experiments. Mean ± SEM. Unpaired t-test, **p<0.01, ***p<0.001.

Moreover, consistent with our results in the AOM/DSS model, we observed smaller tumors in Apc^Min/+^;Itgb7^-/-^ compared to Apc^Min/+^ mice (Fig 3A and 3B), which correlated with a slightly better survival in Apc^Min/+^;Itgb7^-/-^ mice (Fig 3C). Thus, similar to AOM/DSS model, our data suggest that gut-tropic immune cells also promote tumor growth in the spontaneous Apc^Min/+^ intestinal tumor model.

**Fig 4:**
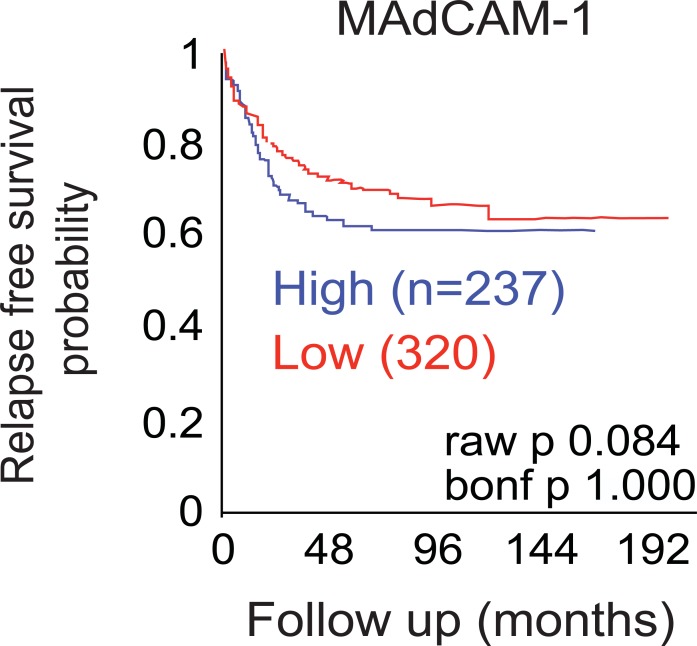
Lower MAdCAM-1 expression is associated with increase survival in CRC patients. Kaplan-Meier analysis showing disease free survival of patients with CRC (*n* = 557) on the basis of *madcam-1* mRNA expression using publicly available data sets (source: AMC onco genomics).

**Fig 5:**
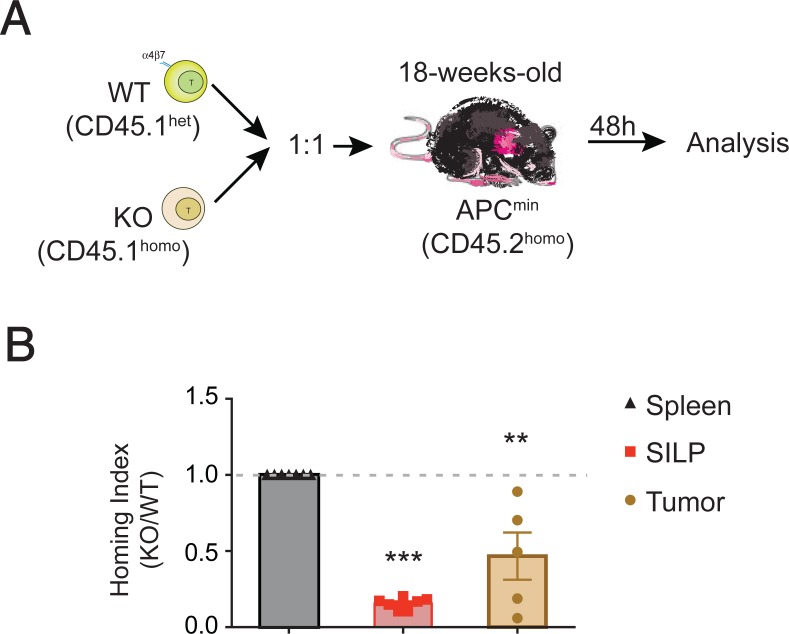
T cells require Itgb7 to home to the intestinal tumors. (A) Congenic WT (CD45.1^het^) and Itgb7^-/-^ (CD45.1^homo^) total T cells were isolated from the spleen and injected intravenously in a 1:1 ratio into 10-week-old (ongoing tumor development) Apc^Min/+^ mice. Twelve hours after the injection, T cell ratios were analyzed in the spleen, small intestine lamina propria (SILP) and tumors. (B) The homing index was calculated as the ratio KO to WT divided by the ratio KO to WT of the input. Data represent two different experiments with 3–4 mice each. Mean ± SEM, ANOVA with Dunnett as post-test, **p<0.01, ***p<0.001.

Survival analysis of publicly available datasets in human CRCs suggests that patients expressing lower levels of MAdCAM-1, the ligand of α4β7, in the tumor have better survival compared to patients expressing higher levels (Fig 4). This suggests that decreased α4β7^+^ cell might be associated with decreased CRC progression in humans. Although the impact of the immune system in colorectal cancer progression is yet poorly understood, it is well appreciated that combination of cell types and immune environment may result in either promotion or inhibition of tumor growth [21]. For example, patients with high expression of Th1 gene cluster had a better prognosis compared to high expression of Th17 cluster which instead had poor prognosis using an unsupervised hierarchical clustering of a set of genes associated with different T cell subsets [22]. Likewise, even if CD4^+^ T cells have been implicated in tumor surveillance, IL-22-producer CD4^+^ T cells (Th22) migrate and infiltrate the tumor to eventually promote stemness and tumorigenic potential [23]. We next attempt to investigate the mechanisms by which Itgb7 may promote tumor growth. Hence, we next sought to investigate whether the reduced tumor volume observed in Apc^Min/+^;Itgb7^-/-^ mice was associated with decrease T cell infiltration within the tumor. To assess whether T cells require Itgb7 to infiltrate the tumor, we performed homing competitive experiments in which congenic total WT and Itgb7^-/-^ T cells were adoptively transferred in a 1:1 ratio into 10 weeks old Apc^Min/+^ mice (Fig 5A). Twelve hours after the injection the Apc^Min/+^ mice were sacrificed and T cells ratios were analyzed. The homing index (the ratio of KO to WT T cells, corrected by the input) was one in the spleen indicating that either WT or Itgb7^-/-^ preferentially populate this organ. As expected, Itgb7^-/-^ T cells home poorly to the SILP compared to WT T cells as seen by a homing index of ~0.2 (Fig 5B). Analysis of T cell infiltrating the tumors revealed that Itgb7^-/-^ T cells were at a disadvantage to WT T cells in homing to the tumors as seen by a homing index of ~0.5 (Fig 5B), indicating that Itgb7 provides T cells a higher capacity to home to intestinal tumors.

Our data suggest that immune cells expressing β7 integrins are not required for tumor initiation in the gut but might promote tumor growth. Therefore, the earliest molecular events initiating the appearance of intestinal tumors might be independent of cell migration to the intestine, at least in a β7 integrin-dependent manner. Supporting this hypothesis, it has been shown that alteration of inflammasome or MyD88 signaling pathways in the intestinal epithelium, but not in hematopoietic-derived cells, results in a higher susceptibility to develop colon cancer [24], and that specific *Apc* mutations in intestinal stem cells (using Lgr5-cre mice) are sufficient to induce rapid tumor development [25].

On the other hand, gut-tropic immune cells are believed to drive intestinal tumor progression by promoting angiogenesis, inflammation and/or proliferation of cancer cells [3, 23, 26]. Therefore, the decreased tumor sizes in Apc^Min/+^;Itgb7^-/-^ as well as in AOM/DSS-treated Itgb7^-/-^ mice could be explained by impaired homing of pro-inflammatory T_EFF_ to the gut. An apparent discrepancy with this hypothesis is the fact that higher numbers of Th1 cells as well as cytotoxic CD8^+^ T cells within tumors correlate with a better prognosis [3]. However, a recent study showed comparable numbers of cytotoxic- and regulatory- CD8^+^ T cells infiltrating the tumor [3], suggesting that regulatory rather than cytotoxic-CD8 T cells account for a better prognosis factor. Indeed, it has been proposed that T_REG_ rather than CD8^+^, and CD45RO^+^ T cell density in the tumor tissues as a stronger prognosis factor in CRC [3]. Of note, location of Foxp3^+^ T_REG_ in the gut might not critically rely on gut-homing receptors [27]. Therefore, the net effect of lacking α4β7 would be an increased T_REG_ / T_EFF_ ratio, which might contribute to decreased inflammation and subsequent intestinal tumorigenesis. In the same line, CD4^+^ T helper subsets might differentially contribute to either tumor initiation, progression or regression. For instance, Th1 seems to be protective as seen by a better survival in patients with higher Th1 numbers [28]. Whereas Th22 cells might promote tumor growth [29]. Hence, it would be interesting to determine whether specific T helper subsets (e.g. Th1) require Itgb7 to modulate tumorigenesis. Experiments in which WT or Itgb7^-/-^ *in vitro* differentiated T helper subsets transferred in mice undergoing intestinal tumorigenesis might provide important insights into this question.

In addition to lymphocytes, some innate immune cells also express α4β7 [30], and future studies will be aimed to dissect the relative contributions of the specific gut-tropic adaptive and innate immune cells subsets in intestinal tumorigenesis. Our findings might have translational implications, as drugs blocking gut-homing receptors such as vedolizumab (anti-α4β7) are currently widely used in IBD patients to ameliorate inflammation, and colon cancer is a potential complication of chronic intestinal inflammation in these patients.
